# Isolation and Identification of Sodium Fluoroacetate Degrading Bacteria from Caprine Rumen in Brazil

**DOI:** 10.1100/2012/178254

**Published:** 2012-07-31

**Authors:** Expedito K. A. Camboim, Arthur P. Almeida, Michelle Z. Tadra-Sfeir, Felício G. Junior, Paulo P. Andrade, Chris S. McSweeney, Marcia A. Melo, Franklin Riet-Correa

**Affiliations:** ^1^Unidade Acadêmica de Medicina Veterinária, Universidade Federal de Campina Grande, 58708 Patos, PB, Brazil; ^2^Laboratório de Fixação Biológica de Nitrogênio, Departamento de Bioquímica e Biologia Molecular, Universidade Federal do Paraná, 80060 Curitiba, PR, Brazil; ^3^Departamento de Genética, Universidade Federal de Pernambuco, 50670 Recife, PE, Brazil; ^4^CSIRO Livestock Industries, Queensland Bioscience Precinct, St Lucia, QLD 4067, Australia

## Abstract

The objective of this paper was to report the isolation of two fluoroacetate degrading bacteria from the rumen of goats. The animals were adult goats, males, crossbred, with rumen fistula, fed with hay, and native pasture. The rumen fluid was obtained through the rumen fistula and immediately was inoculated 100 **μ**L in mineral medium added with 20 mmol L^−1^ sodium fluoroacetate (SF), incubated at 39°C in an orbital shaker. *Pseudomonas fluorescens* (strain DSM 8341) was used as positive control for fluoroacetate dehalogenase activity. Two isolates were identified by 16S rRNA gene sequencing as *Pigmentiphaga kullae* (ECPB08) and *Ancylobacter dichloromethanicus* (ECPB09). These bacteria degraded sodium fluoroacetate, releasing 20 mmol L^−1^ of fluoride ion after 32 hours of incubation in Brunner medium containing 20 mmol L^−1^ of SF. There are no previous reports of fluoroacetate dehalogenase activity for *P. kullae* and *A. dichloromethanicus*. Control measures to prevent plant intoxication, including use of fences, herbicides, or other methods of eliminating poisonous plants, have been unsuccessful to avoid poisoning by fluoroacetate containing plants in Brazil. In this way, *P. kullae* and *A. dichloromethanicus* may be used to colonize the rumen of susceptible animals to avoid intoxication by fluoroacetate containing plants.

## 1. Introduction

The most important group of poisonous plants in Brazil is one that causes “sudden death” during physical effort, responsible for nearly 500 000 cattle deaths each year. It is composed of 13 species of plants, belonging to 3 families: Rubiaceae (*Palicourea marcgravii*, *P. aeneofusca*, *P. juruana*, and *P. grandiflora*); Bignoniaceae (*Pseudocalymma elegans*, *Arrabidaea bilabiata*, and *A. japurensis*); and Malpighiaceae (*Mascagnia rigida*, *M. elegans*, *M. pubiflora*, *M. aff. rigida*, *M. exotropica,* and *M. sepium*) [1–10]. *P. marcgravii* is the most important Brazilian toxic plant and *M. rigida *is the most important toxic plant in Northeastern Brazil [11]. Sodium fluoroacetate was identified as the active principle in *P. marcgravii*, *A. bilabiata* [12, 13], and *M. rigida* [14]. In the other plants the active principle has not been identified, but is probably also sodium fluoroacetate [15]; it disrupts the tricarboxylic acid cycle, being first converted to fluorocitrate, which in turn inhibits the enzymes aconitase and succinate dehydrogenase resulting in citrate accumulation in tissues and plasma, and ultimately causing energy deprivation, and death [16]. Microbial degradation of sodium fluoroacetate is catalyzed by a fluoroacetate dehalogenase, which cleaves the strong carbon-fluorine bond [17], but also cleaves, although less effectively, other bonds such as carbon-chlorine, carbon-bromine and carbon-iodine [18]. Sodium fluoroacetate can also be defluorinated by L-2 haloacid dehalogenases [19]. 

Several soil bacteria have the ability to degrade sodium fluoroacetate. In central Australia, seven fluoroacetate degrading bacteria genera including *Acinetobacter, Arthrobacter, Aureobacterium, Bacillus, Pseudomonas, Weeksella,* and* Streptomyces* were isolated [20]. Recently, seven bacterial strains isolated from Brazilian soil samples have been described, belonging to the genera *Ancylobacter*, *Burkholderia*,* Cupriavidus*,* Paenibacillus*, *Staphylococcus*, *Stenotrophomonas,* and *Ralstonia* [21]. However, a single ruminal bacterial isolate, belonging to the phylum Synergistetes, was reported as being able to degrade sodium fluoroacetate [22, 23]. As the result of our attempt to ascertain whether naturally-occurring fluoroacetate-degrading microorganisms exist in the gastrointestinal systems of animals, we report here the isolation of two new fluoroacetate degrading bacteria from goat rumen.

## 2. Material and Methods

### 2.1. Collection and Samples Processing

The animals were male adult goats, crossbred, with rumen fistula, and fed with hay, native pasture, and water *ad libitum*. They were housed in the Veterinary Hospital of the Universidade Federal de Campina Grande in the State of Paraíba, Northeastern Brazil, and had no access to areas with sodium fluoroacetate-containing toxic plants.

The rumen fluid was obtained through the rumen fistula and immediately inoculated in test tubes.

### 2.2. Bacterial Isolation

Bacterial isolation started by the inoculation of 100 *μ*L of rumen fluid in tubes containing 9 mL of mineral medium (Brunner) added with vitamins (http://www.dsmz.de/microorganisms/medium/pdf/DSMZ_Medium457.pdf) and 20 mmol L^−1^ sodium fluoroacetate (SF) (Sigma-Fluka) as single carbon source. This medium will be here designated as Brunner medium. Samples were incubated at 39°C in an orbital shaker. After 48 hours, 1 mL of the first growth was transferred to test tubes containing 9 mL of Brunner medium and incubated under the same conditions described above.

The SF defluorination was measured with a F^−^ selective electrode (Thermo Electron Corporation) in 24-well plates containing 500 *μ*L of culture and 500 *μ*L of Total Ionic Strength Adjustment Buffer-TISAB (Diaminocyclohexane, sodium chloride, and glacial acetic acid, pH 5.5). The fluoride ion released by the microbial SF degradation was expressed in millimoles, the defluorination rate of 20 mmol L^−1^ corresponding to the release of 20 mmol L^−1^ F^−^.

Samples presenting SF defluorination were cultivated in serial dilutions from 10^−1^ to 10^−9^. To obtain pure colonies the highest dilution that presented SF defluorination was plated on Brunner agar (Brunner medium with agar 1%) and incubated at 39°C for 72 hours. Subsequently, each colony was used to inoculate three test tubes containing 9 mL of Brunner medium, which were monitored for SF defluorination. *Pseudomonas fluorescens* (strain DSM 8341) was used as positive control for fluoroacetate dehalogenase activity, cultivated under the same conditions except that the incubation was at 28°C [24]. Nine mL of Brunner medium without bacteria were incubated under the same conditions to evaluate the sodium fluoroacetate degradation background.

### 2.3. ****16S rRNA Gene Sequence Identification

Genus identification for bacteria displaying defluorination activity was done by polymerase chain reaction (PCR) amplification and sequencing of the 16S rRNA gene. DNA extraction was performed with Brazol (LGC Biotechnology) according to the manufacturer's specifications. 16S rRNA gene was amplified in buffer containing 0.5 *μ*M of 27f and 1492r universal primers [25], 2U of Taq DNA polymerase, 0.2 mM of dNTP and 100 ng of DNA and ultrapure water to a final volume of 20 *μ*L. In the negative control, the DNA volume was substituted by ultrapure water. The amplified products were applied into 1% agarose gel and submitted to electrophoresis. DNA was stained with ethidium bromide and bands visualized with an imaging system (UVP-Bioimaging Systems).

The sequencing reaction was performed with BigDye kit according to manufacturer's recommendations (Applied Biosystems) and the product sequenced in the Genetic Analyzer 3500 XL sequencer (Applied Biosystems).

### 2.4. Sequence Analysis and Phylogram

16S rRNA gene sequences were assembled with the CAP3 Sequence Assembly Program (http://pbil.univ-lyon1.fr/cap3.php). DNA sequences were analyzed by basic local alignment search tool (BLAST) available on the website of the National Center for Biotechnology Information (NCB—http://www.ncbi.nlm.nih.gov/BLAST). Species identification was based on maximum score, identity and coverage values. The Greengenes database and workbench were used to corroborate species identification (http://greengenes.lbl.gov). The Bootstrap consensus tree was generated with MEGA5 software using the neighbor-joining statistical method (http://www.megasoftware.net/mega.php) [26]. 

## 3. Results and Discussion

The 16S rRNA gene sequences from the two fluoroacetate degrading isolates ECPB08 (JQ345720) and ECPB09 (JQ345721) were similar to the 16S rRNA genes from *Pigmentiphaga* and *Ancylobacter *species, respectively (Table 1). On the basis of score value alone, the *P*. *kullae* sequence was the most similar (score = 2237) to ECPB08, although other 16S rRNA sequences had the same coverage and identity parameter values. Similarly, the 16S rRNA sequences from *Ancylobacter vacuolatus* and *Ancylobacter sp.* strain WPCB135 were the most similar (score 2388) to ECPB09, but the differences in alignment parameters for other *Ancylobacter* 16S rRNA sequences were only marginal. Consequently, it was not possible to infer from the score values which species would be phylogenetically the closest to ECPB09 (JQ345721).

**Figure 1:**
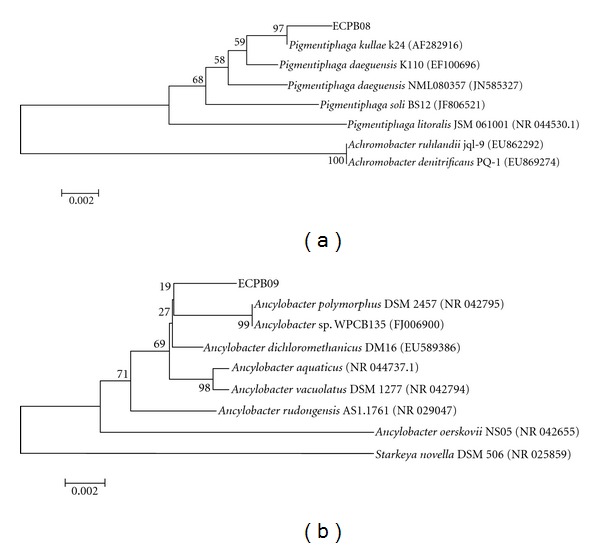
Phylogenetic tree based on 16S rRNA sequences by Maximum Parsimony analysis. ECPB08 (JQ345720) and ECPB09 (JQ345721) are the codes for the isolates. In parentheses are the GenBank codes. The relationship between ECPB08 (JQ345720) (a), ECPB09 (JQ345721) (b) related taxa, and the outgroups *Achromobacter ruhlandii, A. denitrificans,* and *Starkeya* *novella* is shown. The bootstrap consensus tree inferred from 1000 replicates is taken to represent the evolutionary history of the taxa analyzed. Scale of 0.002: evolutionary distance.

Comparative sequence analysis using global alignment confirmed that the isolates ECPB08 (JQ345720) and ECPB09 (JQ345721) were phylogenetically closely related to the genera *Pigmentiphaga* and *Ancylobacter*, respectively (Figure 1). Indeed, the global alignment corroborates the taxonomic inference based on the 16S rRNA local alignment results for ECPB08 (JQ345720), indicating that this isolate is closely related to *P. kullae* strain K24 (Figure 1(a)), and is supported by previous results on the taxonomy of this genus [27, 28]. Moreover, the global alignment allowed a better estimative of taxon position for ECPB09 (JQ345721), suggesting its close relationship with *A. polymorphus* (Figure 1(b)). Although the upper part of the tree in Figure 1(b) has low bootstrap values, the whole dendrogram is consistent with the results presented by Firsova et al. [29] for the genus *Ancylobacter*. 

The genus *Pigmentiphaga* was initially proposed by Blümel et al. [30] with *Pigmentiphaga kullae* (strain K24) as its unique specie, which degrades xenobiotic compounds. In 2007, Yoon et al. [31] described a new member of the genus, *Pigmentiphaga daeguensis* (strain K110T), isolated from wastewater collected from a dye works in Korea. Later, Chen et al. [27] and Lee et al. [28] isolated *Pigmentiphaga litoralis* (strain JSM 061001) and *P. soli *from a tidal flat sample in the South China Sea and from soil in South Korea, respectively.

Raj and Maloy [32] proposed the replacement of the genus name *Microcyclus* by *Ancylobacter*, with a single species, *A. aquaticus* [33]. Since then, five new species have been described, the latest being *Ancylobacter dichloromethanicus* (strain DM16), isolated from contaminated soil [29]. This species uses dichloromethane, methanol, formate, and formaldehyde polycarbonate compounds as carbon and energy sources. Enzymatic analysis showed that it contains a GSH-dependent dichloromethane dehalogenase.

**Figure 2:**
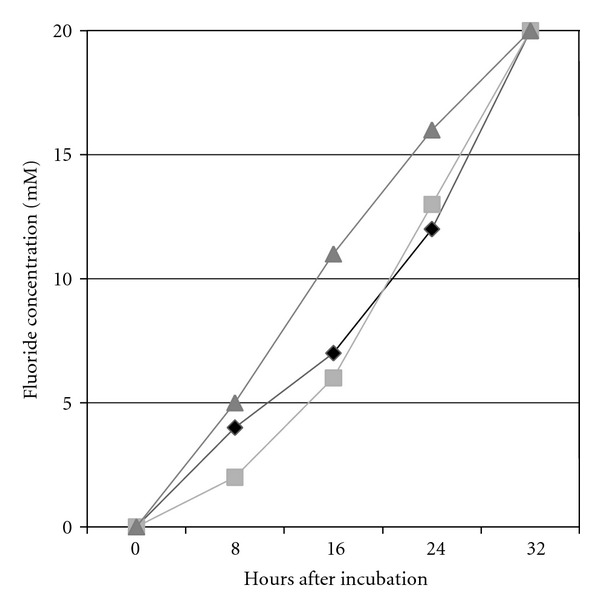
Sodium fluoroacetate degradation rate by bacteria isolated from caprine rumen. Isolate ECPB08 (light grey square), isolate ECPB09 (black rhombus), and *P. fluorescens* (dark grey triangle, positive control).

Both ECPB08 (JQ345720) and ECPB09 (JQ345721) grew in culture medium containing SF as the sole carbon source. These bacteria displayed SF degradation activity, releasing 20 mmol L^−1^ of fluoride ion after 32 hours of incubation in Brunner medium with 20 mmol L^−1^ of SF (Figure 2). These results are similar to those reported by Davis et al. [34] for *Burkholderia *sp. The *Pseudomonas fluorescens* control strain (DSM 8341) reached the same level of defluorination, and there was no release of fluoride ions when the Brunner medium was incubated without bacteria, due to the stability of the strong carbon-fluorine bond in the fluoroacetate [17]. There are no previous reports of fluoroacetate dehalogenase activity for any *Pigmentiphaga* or *Ancylobacter* species.

The defluorination ability of both isolates can be due to the expression of a fluoroacetate dehalogenase gene or to another unspecific dehalogenase. Although for *Pigmentiphaga* no dehalogenase gene has yet been described, a dichloromethane dehalogenase sequence from *A. dichloromethanicus* is deposited in GenBank [29]. Liu et al. [35] confirmed that fluoroacetate dehalogenase degrades other halogenated compounds such as chloroacetate, bromoacetate, iodoacetate, and dichloroacetate, by a cross-adaptation mechanism [36]. Similar results were obtained by Donnelly and Murphy [24] who found a fluoroacetate dehalogenase able to degrade chloroacetate, bromoacetate, and ethyl fluoroacetate. Alternatively, sodium fluoroacetate was defluorinated by L-2 haloacid dehalogenase [18].

Sodium fluoroacetate degrading bacteria, mostly *Bacillus* and *Flavobacterium* species, were isolated from soil in Australia, even in the absence of sodium fluoroacetate in the environment [37]. In this study, two *Pigmentiphaga *and* Ancylobacter* species were isolated from goat rumen, although the animals were not fed plants that produce this component. This finding suggests that the fluoroacetate dehalogenase gene is rather ubiquitous and that its expression may represent a selective advantage for microorganism, possible due to the ability of the enzyme to degrade other related compounds, as mentioned before.

On the other hand, it has been reported that animals grazing in areas where *Mascagnia rigida* is present are more resistant to poisoning than those kept in areas without these plants [6]. Recent results of our research group demonstrated that resistance to *M. rigida *poisoning can be induced by the administration of repeated low nontoxic doses of the plant or by transfaunation of rumen fauna from resistant to susceptible animals (unpublished data). These results suggest that either changes in rumen microflora or sustained high expression levels of the SF dehalogenase gene from the endogenous rumen microbiota are responsible for this tolerance. 

Control measures to prevent plant intoxication, including use of fences, herbicides, or other methods of eliminating poisonous plants, have been unsuccessful to avoid poisoning by fluoroacetate containing plants in Brazil [38]. An alternative way would be the microbial detoxification of plants by inoculation of fluoroacetate degrading bacteria into the rumen. This strategy was used by Gregg et al. [39] who inoculated in the rumen a genetically modified strain of *Butyrivibrio fibrisolvens* with a gene encoding fluoroacetate dehalogenase from *Moraxella* species, which was efficient to prevent fluoroacetate poisoning in sheep. In an alternative approach, the two isolates, *Pigmentiphaga *sp. and *Ancylobacter *sp. are candidates to be used in colonizing the rumen to avoid intoxication by fluoroacetate containing plants. 

## Figures and Tables

**Table 1:** Local alignment results for fluoroacetate-degrading bacterial isolates 16S rRNA sequences. Sequences were compared to those deposited at the National Center for Biotechnology Information using the regular BlastN algorithm available at http://www.ncbi.nlm.nih.gov/BLAST. Organisms with sequences similar to those of isolates ECPB08 (JQ345720) and ECPB09 (JQ345721) are listed on the second column. Values for the parameters total score, query coverage, and maximum identity are displayed in columns 3 to 5. GenBank access codes are displayed in parentheses.

Isolate (acc. no.)	Species/strain (accession no.)	Total score	Query coverage (%)	Max identity (%)
ECPB08 (JQ345720)	*Pigmentiphaga kullae* strain K24 (NR_025112.1)	2237	100	98
	*Pigmentiphaga daeguensis* strain K110 (NR_044082.1)	2215	100	98
	*Pigmentiphaga daeguensis* strain NML080357 (JN585327.1)	2197	100	97
	*Pigmentiphaga litoralis* strain JSM 061001 (NR_044530.1)	2152	99	97
ECPB09 (JQ345721)	*Ancylobacter* sp. WPCB135 (FJ006900.1)	2388	99	99
	*Ancylobacter vacuolatus* strain DSM 1277 (NR_042794.1)	2388	99	99
	*Ancylobacter dichloromethanicus* strain DM16 (EU589386.1)	2387	99	99
	*Ancylobacter polymorphus* strain DSM 2457 (NR_042795.1)	2372	99	99
	*Ancylobacter rudongensis* strain AS1.1761 (NR_029047.1)	2370	99	99
	*Ancylobacter polymorphus* T10AII (GQ921957.1)	2358	98	99
	*Ancylobacter aquaticus* (NR_044737.1)	2351	99	98
	*Ancylobacter oerskovii* strain NS05 (NR_042655.1)	2341	99	98
